# Identification of the phosphatase essential for riboflavin biosynthesis in *Aquifex aeolicus*

**DOI:** 10.1016/j.jbc.2025.108443

**Published:** 2025-03-25

**Authors:** Zoe A. Hoffpauir, Audrey L. Lamb

**Affiliations:** Department of Chemistry, 1 UTSA Circle, University of Texas at San Antonio, San Antonio, Texas, USA

**Keywords:** riboflavin, phosphatase, hydrolase, thermophile, isomerase, mutase, lumazine, enzyme structure

## Abstract

The riboflavin biosynthetic pathway uses dedicated enzymes that function exclusively for riboflavin production. Indeed, the pathway is fully annotated, with the exception of an unknown phosphatase that catalyzes the dephosphorylation of 5-amino-6-ribitylamino-pyrimidinedione 5′-phosphate (ARAPDP) to generate 5-amino-6-ribitylamino-pyrimidinedione (ARAPD), which is the substrate for the penultimate enzyme of the pathway, lumazine synthase. Whereas nonspecific phosphatases from the haloacid dehalogenase superfamily capable of catalyzing the dephosphorylation of ARAPDP have been reported for *Bacillus subtilis, Escherichia coli,* and *Arabadopsis thaliana*, we hypothesized that a specific phosphatase may carry out this reaction. Using an anaerobic activity-based screen, two phosphatases from *Aquifex aeolicus* were identified that dephosphorylate ARAPDP, but only one reconstitutes riboflavin production in a one-pot experiment with the other four enzymes of riboflavin biosynthesis. The first enzyme, annotated as an IMP, is nonspecific, and indiscriminately dephosphorylates ARAPDP along with ribulose 5-phosphate and NADPH, two required substrates of riboflavin biosynthesis. The second enzyme, a histidine family phosphatase, only dephosphorylates ARAPDP in the one-pot experiment thus facilitating riboflavin formation. The structures of both enzymes were determined by X-ray crystallography to reveal the vastly different folds capable of performing the ARAPDP dephosphorylation chemistry. This work has impact both for the production of riboflavin by microbial fermentation and for antimicrobial drug design.

Riboflavin, or vitamin B2, is the universal precursor to flavin mononucleotide (FMN) and flavin adenine dinucleotide (FAD), essential cofactors across all kingdoms of life (1, 2). Although humans and other animals obtain riboflavin through dietary sources, plants and bacteria synthesize riboflavin using specialized enzymes that humans lack. Indeed, the enzymes of riboflavin biosynthesis are an attractive target for the development of novel antimicrobials as many pathogenic bacteria lack efficient riboflavin uptake machinery (3, 4, 5), thus relying on endogenously synthesized riboflavin.

The riboflavin biosynthetic pathway is composed of two distinct branches that converge at the penultimate step of riboflavin formation (Fig. 1). The initial substrates of the individual branches are GTP and D-ribulose 5-phosphate (Ru5P). The A branch is initiated by GTP cyclohydrolase II (RibA) catalyzing the conversion of GTP to 2,5-diamino-6-ribosylamino-pyrimidinone 5′-phosphate (DARP), which is subsequently converted to 5-amino-6-ribitylamino-pyrimidinedione 5′-phosphate (ARAPDP) by the bifunctional pyrimidine deaminase/reductase (RibD). Meanwhile, the B branch is initiated by the conversion of Ru5P to 3,4-dihydroxy-2-butanone 4-phosphate (DHBP) by 3,4-dihydroxy-2-butanone 4-phosphate synthase (RibB). Lumazine synthase converts one molecule of ARAPD and one molecule of DHBP to 6,7-dimethyl-8-ribitlyllumazine (lumazine). Two molecules of lumazine are required by riboflavin synthase to generate one molecule of riboflavin and regenerate one molecule of ARAPD.

**Figure 1:**
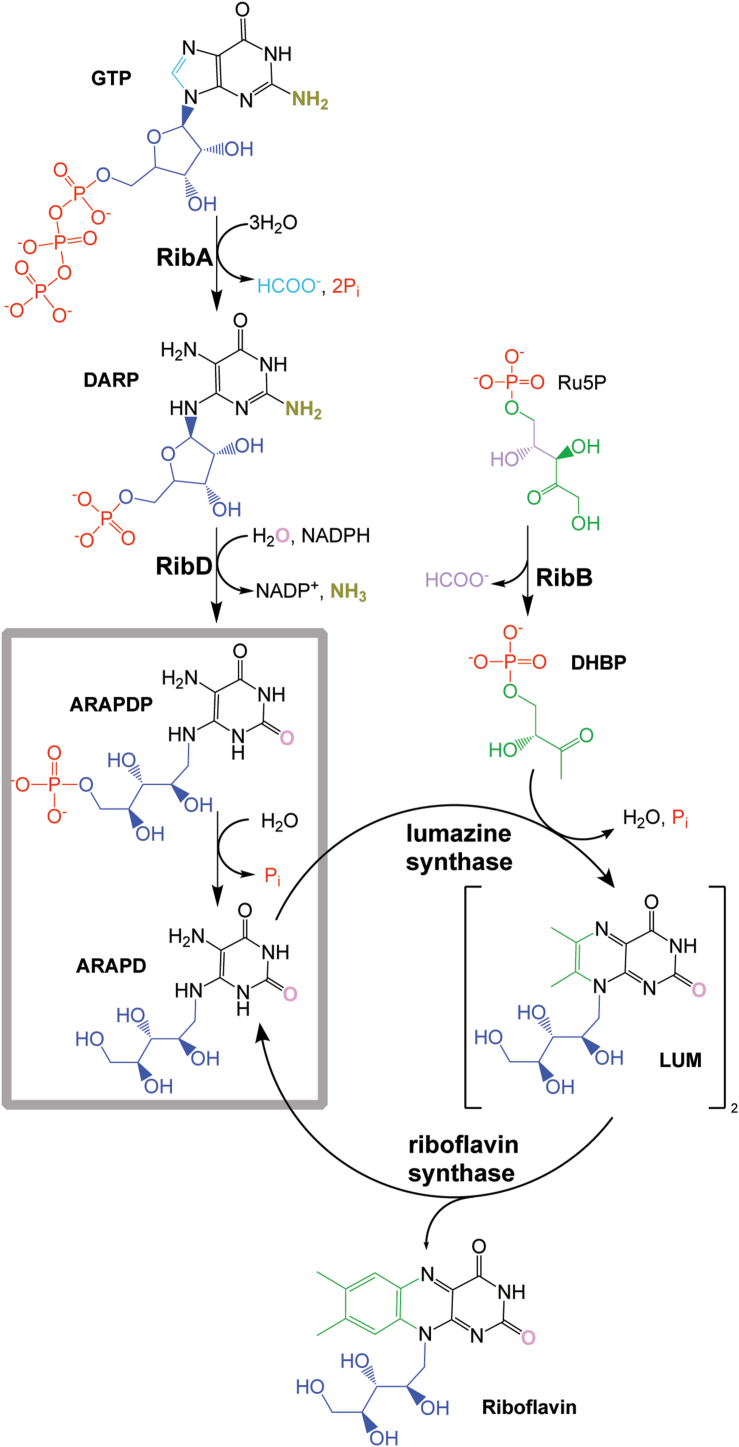
**Riboflavin biosynthetic pathway with dedicated enzymes noted**. Highlighted by the *gray box* is the dephosphorylation step of interest that lacks a dedicated phosphatase. ARAPD, 5-amino-6-ribitylamino-pyrimidinedione; ARAPDP, 5-amino-6-ribitylamino-pyrimidinedione 5′-phosphate; DARP, 2,5-diamino-6-ribosylamino-pyrimidinone 5′-phosphate; DHBP, 3,4-dihydroxy-2-butanone 4-phosphate; LUM, 6,7-dimethyl-8-ribitlyllumazine; RibA, GTP cyclohydrolase II; RibB, 3,4-dihydroxy-2-butanone 4-phosphate synthase; RibD, bifunctional pyrimidine deaminase/reductase; Ru5P, D-ribulose 5-phosphate.

The riboflavin biosynthetic pathway is well-studied with dedicated enzymes to perform complex chemical transformations (6, 7, 8), with the exception of an unknown phosphatase that catalyzes the dephosphorylation of ARAPDP, the product of RibD, to generate 5-amino-6-ribitylamino-pyrimidinedione (ARAPD), the substrate for lumazine synthase. Nonspecific phosphatases capable of catalyzing the dephosphorylation of ARAPDP have been reported for *Bacillus subtilis* (9)*, Escherichia coli* (10), and *Arabidopsis thaliana* (11), but a dedicated phosphatase has yet to be identified. All of the reported ARAPDP phosphatases belong to the haloacid dehalogenase (HAD) superfamily of phosphatases.

With the goal of identifying a dedicated phosphatase to complete the riboflavin biosynthetic pathway, we screened phosphatases from *Aquifex aeolicus*, a hyperthermophile with a relatively small genome from which the riboflavin biosynthetic enzymes have been studied (8, 12, 13, 14, 15). The thermal stability of the putative phosphatases was a key element for streamlining the screening, as a simple, crude purification was afforded by heating the lysates to denature the proteins of the heterologous production system (*E. coli*). Two phosphatases were identified that perform the necessary reaction, while only one facilitates riboflavin formation when combined with the other enzymes of the riboflavin pathway and their initial substrates, Ru5P, GTP, and NADPH. Neither of these two phosphatases belongs to the HAD superfamily.

## Results

### The phosphatases of the *Aquifex aeolicus* genome

Previously reported nonspecific ARAPDP phosphatases from *E. coli* (10), *B*. *subtilis* (9), and *A. thaliana* (11) belong to the HAD superfamily, so all *A. aeolicus* genes annotated as HAD or HAD-like hydrolases were chosen for screening. The short consensus sequences that are characteristic of HAD hydrolases (11, 16) were identified in the *A. aeolicus* proteome using a Python script (Script S1). In addition, 20 genes in the *A. aeolicus* genome are annotated as phosphatases. Of those putative phosphatases, 19 had a predicted molecular weight of 40 kDa or less. This molecular weight cutoff was chosen for two reasons: (1) previously determined nonspecific phosphatases that dephosphorylate ARAPDP are smaller than 40 kDa, and (2) phosphatases larger than 40 kDa are likely to have additional domains with other functions. The 40 kDa molecular weight threshold only eliminated one putative phosphatase (PP) from screening, which has a predicted molecular weight of 42.1 kDa and is annotated as fructose-1,6-bisphosphate aldolase/phosphatase.

The annotated phosphatases and HAD hydrolases under 40 kDa (23 in total) in the *A. aeolicus* genome were produced for screening of ARAPDP phosphatase activity. Overexpression constructs were generated, and the proteins were heterologously overproduced in *E. coli.* A crude purification protocol that takes advantage of the heat stability of the *A. aeolicus* enzymes was developed. Expression and relative purity were monitored by SDS-PAGE (Fig. S1). Of the 23 proteins selected, 22 expressed sufficiently for screening. Putative phosphatase 13 (PP13), annotated as the integral membrane protein undecaprenyl-diphosphatase (UppP), was not produced in the heterologous system and was the only putative phosphatase that was not screened in the assay. Table S1 summarizes the enzymes selected.

### Detection of ARAPDP phosphatase activity

**Figure 2:**
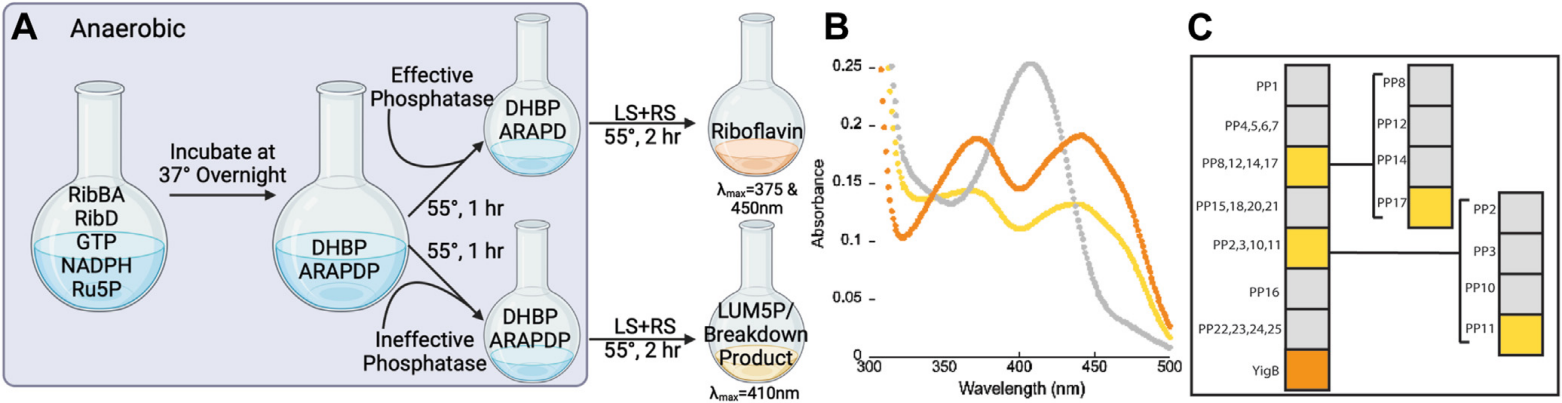
**Phosphatase activity screening assay.***A*, the workflow. Steps highlighted by the *graybox* were performed anaerobically. The figure was generated using BioRender https://BioRender.com/r20n342. *B*, representative results from assay screen. The *gray* spectrum (λ_max_ = 410 nm) results from the accumulation of lumazine, lumazine5P, and a breakdown product of ARAPD in the presence of an ineffective phosphatase. The *yellow* spectrum (λ_max_ = 375 nm and 450 nm) was produced from hits from the screen showing the accumulation of riboflavin. The *orange* spectrum was produced from the positive control, EcYigB. *C*, screen results. The *orange box* is the YigB positive control. *Yellow boxes* denote reactions that produced a riboflavin signal and therefore contained all enzymatic steps of the riboflavin biosynthetic pathway, including an effective phosphatase. *Gray boxes* show ineffective phosphatase activity. The *left boxes* show the multiphosphatase cocktails, whereas the *middle* and *right boxes* show the particular enzymes in the cocktails that demonstrate ARAPDP phosphatase activity. ARAPD, 5-amino-6-ribitylamino-pyrimidinedione; ARAPDP, 5-amino-6-ribitylamino-pyrimidinedione 5′-phosphate.

To identify a phosphatase capable of dephosphorylating ARAPDP and thus connecting the two arms of the riboflavin biosynthetic pathway, it was necessary to simultaneously generate both ARAPDP, the product of the RibA and RibD reactions, and DHBP, the product of the RibB reaction. The initial cocktail contained the *A. aeolicus* enzymes RibBA (the naturally occurring fusion of RibA and RibB), RibD, ribulose 5-phosphate, GTP, and NADPH in an anaerobic environment, which was allowed to incubate at 37 °C overnight (Fig. 2*A*). It is necessary to perform this reaction anaerobically, because ARAPDP is unstable in oxygen and will break down, as previously described (17). To increase the throughput of the activity screening, up to four PPs were added. The mixture was incubated at 55 °C for 1 h. While a phosphatase in the cocktail that dephosphorylates DHBP would give a false negative, 3,4-dihydroxy-2-butanone (DHB) has no known physiological purpose (18), so an enzyme evolved to synthesize DHB from DHBP would be unlikely. A nonspecific phosphatase capable of dephosphorylating DHBP would lack efficiency; therefore, sufficient DHBP would remain in the assay for the production of riboflavin in the screen. The phosphatase YigB from *E. coli* that was previously shown to have ARAPDP phosphatase activity served as a positive control. YigB is considered nonspecific since gene deletion did not impact growth of *E. coli* in the absence of riboflavin (10).

Lumazine synthase (RibH) and riboflavin synthase (RibE) were added, and riboflavin production was monitored after 2 h. The readout of the assay is an absorbance spectrum from 300 to 500 nm, monitoring for characteristic maxima at 410 nm for lumazine or 375 nm and 450 nm for riboflavin. It should be noted that several species absorb similarly to lumazine. ARAPD(P) breaks down significantly and the breakdown product absorbs at 410 nm (17). Similarly, lumazine synthase accepts ARAPDP as a substrate to form lumazine 5-phosphate (lumazine5P), which also absorbs at 410 nm; however, lumazine5P is not a substrate for riboflavin synthase to make FMN (FMN or riboflavin 5-phosphate) (19). Thus, both the ARAPD(P) breakdown products and lumazine5P contribute to the signal seen at 410 nm in the absence of an effective phosphatase (Fig. 2*B*) (9, 10, 11). Riboflavin formation was monitored at 470 nm without interference from breakdown products that absorb at 410 nm (8). If a cocktail of phosphatases demonstrated riboflavin production, the enzymes of the cocktail were then screened individually. Of the 22 phosphatases screened, two were identified that, when combined with the remainder of the riboflavin pathway, produced riboflavin (Fig. 2*C*). These two enzymes were PP11, which is annotated as an myo-inositol-1(or 4)-monophosphatase (IMP), and PP17, a histidine family phosphatase (HFP).

### Determination of phosphatase specificity

**Figure 3:**
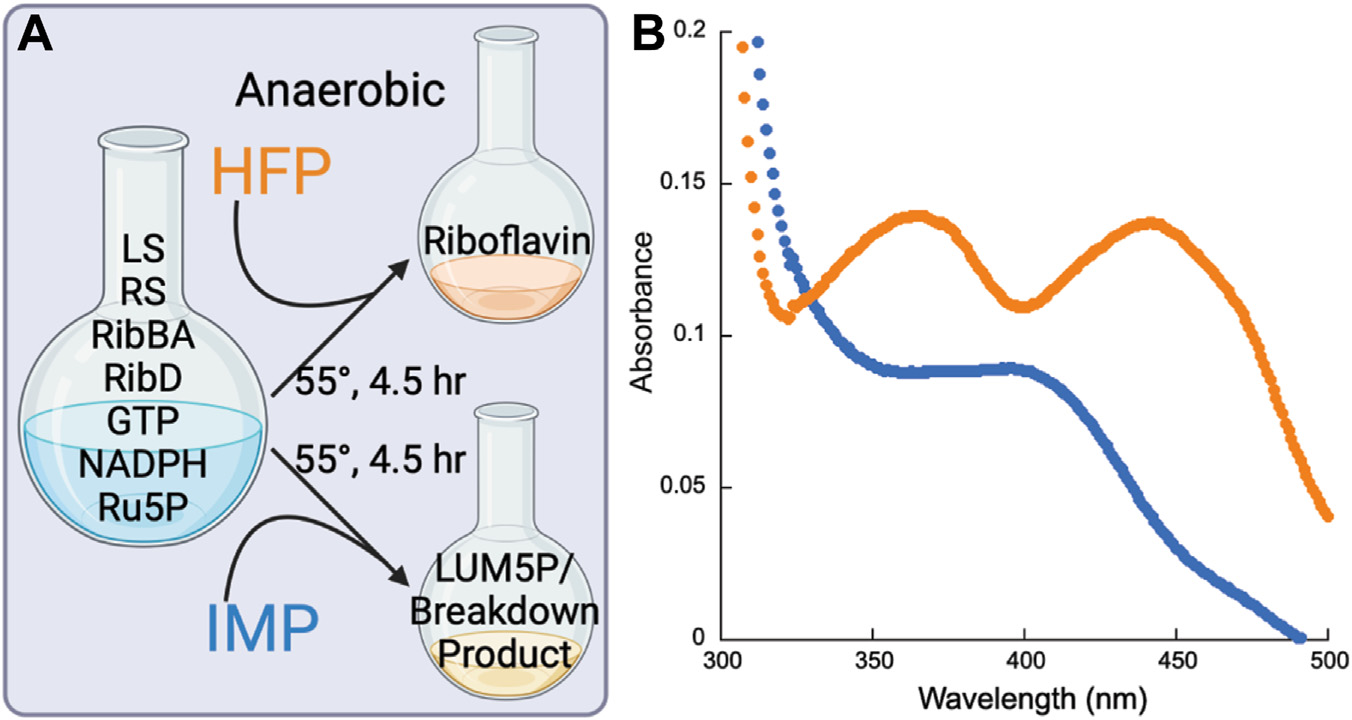
**One-pot reaction.***A*, workflow. The figure was generated using BioRender https://BioRender.com/e64e561. *B*, representative plot of riboflavin biosynthesis with added phosphatases. HFP facilitates riboflavin formation as seen by the characteristic riboflavin double peaks at 360 and 445 nm (*orange*). IMP does not facilitate riboflavin formation under the same conditions (*blue*). HFP, histidine family phosphatase.

The screening assay was designed such that the substrate ARAPDP was in excess when the phosphatases were added, allowing even nonspecific enzymes to convert ARAPDP to ARAPD. However, in the full pathway, there are a variety of physiologically significant phosphorylated molecules that may serve as potential substrates for a nonspecific enzyme (ribulose 5-phosphate, GTP, NADPH, and DARP). Therefore, nonspecific phosphatase activities may compete with the ARAPDP phosphatase activity. For example, if an effective phosphatase in the screening assay also dephosphorylated NADPH with comparable activity, the amount of riboflavin produced would be reduced. Therefore, a new assay was designed to determine the substrate specificity of IMP and HFP (Fig. 3*A*). In this assay, all of the riboflavin biosynthetic enzymes (RibBA, RibD, lumazine synthase, and riboflavin synthase) were mixed with the three necessary substrates (GTP, NADPH, and ribulose 5-phosphate) in the presence of one of the effective phosphatases from the screening assay and the absorbance of products formed was monitored from 300 to 500 nm (Fig. 3*B*). Only HFP but not IMP led to riboflavin formation, implying that IMP acted indiscriminately with the other phosphorylated substrates.

### Determination of competing activities

**Figure 4:**
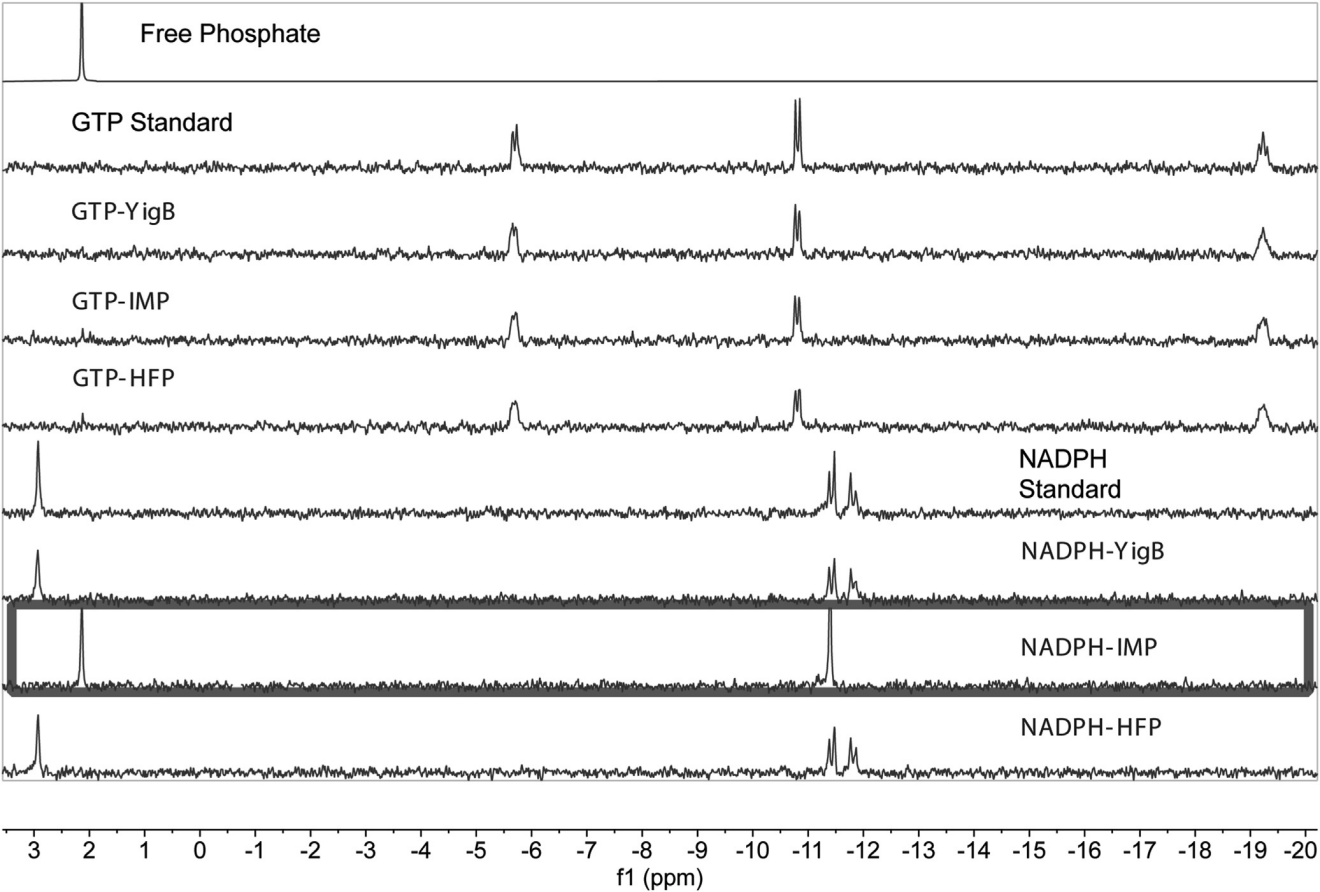
**^31^-Phosphorus NMR assay of phosphatase activities of IMP and HFP against GTP and NADPH.** Neither IMP or HFP dephosphorylate GTP. IMP dephosphorylates NADPH (*box*) while HFP does not. IMP and HFP reactions were incubated at 55 °C for 1 h whereas YigB was incubated for 1 h at 37 °C to account for differing enzyme stabilities (IMP and HFP are from a hyperthermophile; YigB is from *Escherichia coli*). HFP, histidine family phosphatase.

**Figure 5:**
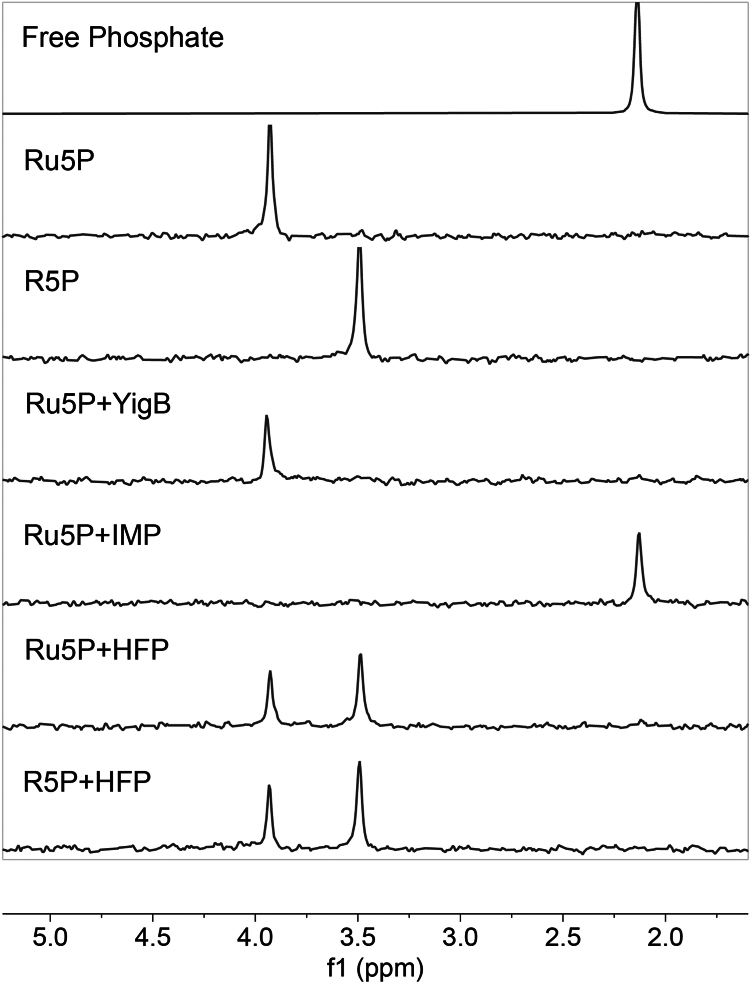
**Phosphatase activities of HFP and IMP against Ru5P using ^31^P-NMR**. Standards show phosphorus chemical shifts of 2.1 ppm (free phosphate), 3.9 ppm (Ru5P), and 3.5 ppm (R5P). In the Ru5P + IMP sample, the sugar phosphate is dephosphorylated, generating free phosphate. In the Ru5P + HFP sample, Ru5P is converted to R5P, establishing an equilibrium of 39.7% to 60.3% Ru5P and R5P, respectively. YigB does not dephosphorylate Ru5P. The addition of R5P to HFP demonstrates the reversibility of the Ru5P/R5P isomerase activity as the same equilibrium is established by starting with either Ru5P or R5P as substrate. HFP, histidine family phosphatase; Ru5P, D-ribulose 5-phosphate; R5P, D-ribose 5-phosphate.

Both IMP and HFP were further purified for characterization (Fig. S2). Phosphorus NMR was used to determine if IMP or HFP dephosphorylate the substrate inputs of the riboflavin biosynthetic pathway: GTP, NADPH, and Ru5P. Neither IMP nor HFP dephosphorylated GTP under conditions similar to those used in the riboflavin biosynthesis assay (Fig. 4): no free phosphate is formed and the GTP remains after incubation with the enzymes for 1 h time at 55 °C. However, at high enzyme concentrations and with extended time (24 h), both IMP and HFP dephosphorylate GTP (Fig. S3). In contrast, IMP dephosphorylated NADPH based on the formation of a free phosphate peak at 2.1 ppm and the decay of NADPH signals between −11.3 to −11.9 ppm into a single peak at −11.4 (Fig. 4). In addition, IMP dephosphorylated ribulose 5-phosphate, as the characteristic Ru5P peak at 3.9 ppm was converted to a free phosphate peak at 2.1 ppm (Fig. 5). Therefore, IMP dephosphorylates both NADPH and Ru5P, making these substrates unavailable for RibBA and RibD, respectively, and thereby explaining why IMP does not facilitate riboflavin formation in the one-pot reaction.

HFP did not dephosphorylate ribulose 5-phosphate; however, phosphorus NMR demonstrated that the sample had isomerase activity interconverting ribulose 5-phosphate and ribose 5-phosphate (Fig. 5), which was confirmed by ^13^C-NMR (Fig. S4). This isomerase activity did not prevent the riboflavin formation in the one-pot reaction. Indeed, the reaction is reversible, establishing an equilibrium mixture of 39.7% ketose (ribulose) and 60.3% aldose (ribose) stereoisomer 5-phosphates in less than 10 min (Fig. S5).

Ribose 5-phosphate isomerases (RpiA or RpiB) of the pentose phosphate pathway demonstrate high turnover numbers and catalytic efficiencies. Indeed, *E. coli* RpiA has a turnover number of 2100 s^−1^ and a catalytic efficiency of 680,000 M^−1^s^−1^ (20). Therefore, trace amounts of contaminant *E. coli* RpiA or RpiB in the sample could account for the isomerase activity. In order to obtain a sample of the highest purity possible, an HFP construct was generated bearing a histidine tag for purification by affinity chromatography. Following purification using affinity chromatography, gel filtration, heat (90 °C for 2 min) followed by centrifugation to remove precipitated proteins, the sample isomerizes Ru5P, although equilibrium is reached much more slowly (>18 h as opposed to <10 min), likely due to less contaminating enzyme present in this sample (Fig. S6). As a final control, five PP samples were selected at random and assayed for isomerase activity. Four of the five samples demonstrated isomerase activity (Fig. S7), a clear indication that a hardy and efficient *E. coli* ribose 5-phosphate isomerase is a common contaminate. The presence of *E. coli* RpiA in the HFP sample was confirmed by mass spectrometry (Fig. S8). While the IMP sample did not demonstrate the isomerase activity directly monitored in the ^31^P-NMR experiment, the isomerization equilibrium was not detected because the sugar phosphate was dephosphorylated.

### Structure determination of IMP

**Figure 6:**
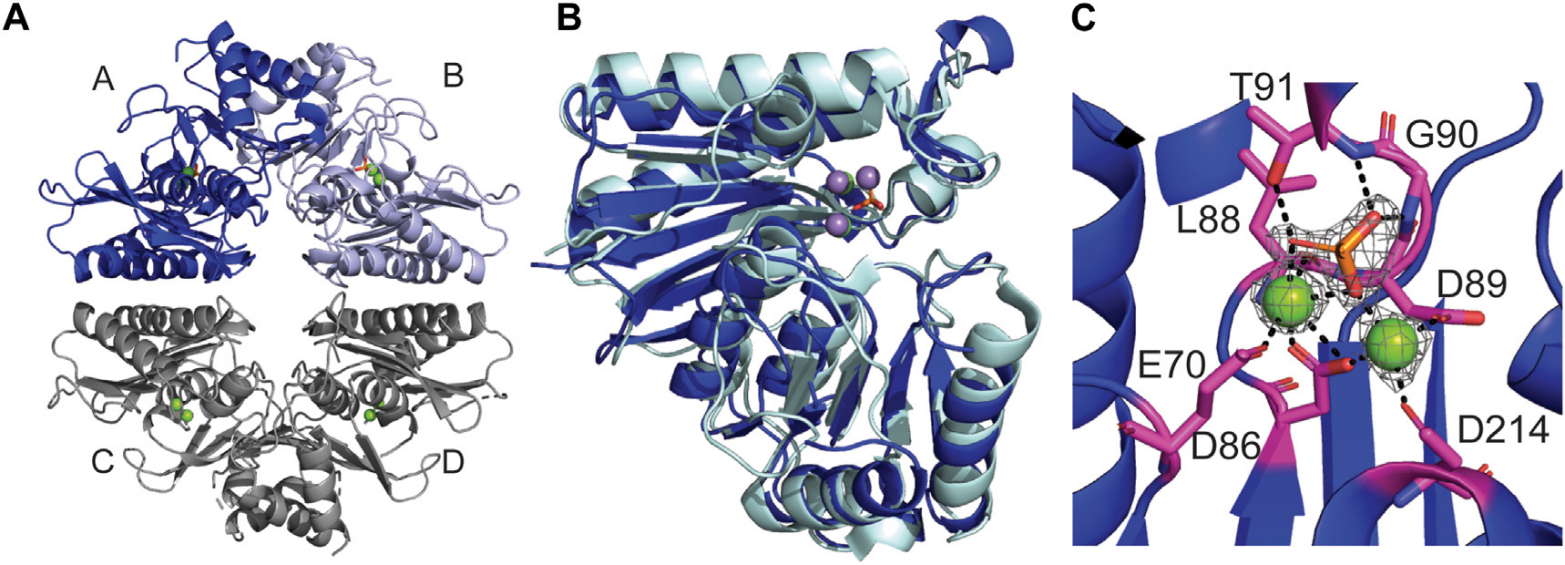
**Structure of IMP.***A*, the asymmetric unit of IMP is composed of A-B (*dark blue* and *light blue*, respectively) and C-D (*dark gray*) homodimers. *B*, superposition of IMP (*dark blue*) with the human myo-inositol monophosphatase (*pale cyan*) (PDB: 6GJ0). Whereas 6GJ0 has three manganese modeled into the active site (*purple spheres*), IMP has two magnesium atoms (*green spheres*) and a phosphate. *C*, active site coordination of residues in *magenta* with magnesium atoms and phosphate. Simulated annealing composite omit map with electron density of ligands contoured to 2 σ. PDB, Protein Data Bank.

IMP is annotated as IMP and has a crystal structure deposited to the Protein Data Bank (PDB) (accession code 2PCR) by the RIKEN Structural Genomics/Proteomics Initiative that lacks an accompanying manuscript. The 2.6 Å deposited structure 2PCR was used as a model for molecular replacement. The structure determined here was solved to 1.75 Å with two homodimers in the asymmetric unit (Fig. 6*A*). Inositol monophosphatases are reported to fold as a α/β/α/β/α penta-layered sandwich (21) that assemble as homodimers (22). IMP displays that standard fold and PDBePISA calculates an interface surface area of greater than 1700 Å^2^ between the A and B chains, consistent with stable dimer formation. In addition, the estimated molecular weight in solution was that of a dimer as determined by preparative gel filtration chromatography during purification. Although inositol monophosphatases are generally reported to be magnesium dependent, a variety of metals, including zinc and manganese, support catalysis (23). Density was observed in the active site for metal ions, but no metals were added during purification or crystallization; therefore, these ions were derived from the heterologous enzyme production in *E. coli.* Inductively coupled plasma mass spectrometry (ICPMS) was used to unambiguously determine that magnesium is bound to the protein. Whereas reported myo-inositol phosphatase structures have three metals modeled in the active site (22, 23, 24), the electron density in the IMP active site only supported modeling of two magnesium ions (Fig. 6*C*). No metals or ligands were modeled into the active site of 2PCR, the previously deposited structure of IMP, likely as a result of the lower resolution. IMP is part of a large protein superfamily with many reported structures that have a highly homologous fold. Indeed, PDBeFOLD calculates that there are more than 50 unique PDB entries with an RMSD 1.03 to 1.97 Å for alignment of 199 to 244 C-alphas. A notable human homologue of IMP is the putative target for lithium therapy (22, 23, 24) which has an RMSD of 1.60 Å (PDB: 6GJ0) and a sequence similarity of 50% and identity of 32% (Fig. 6*B*).

### Structure determination of HFP

**Figure 7:**
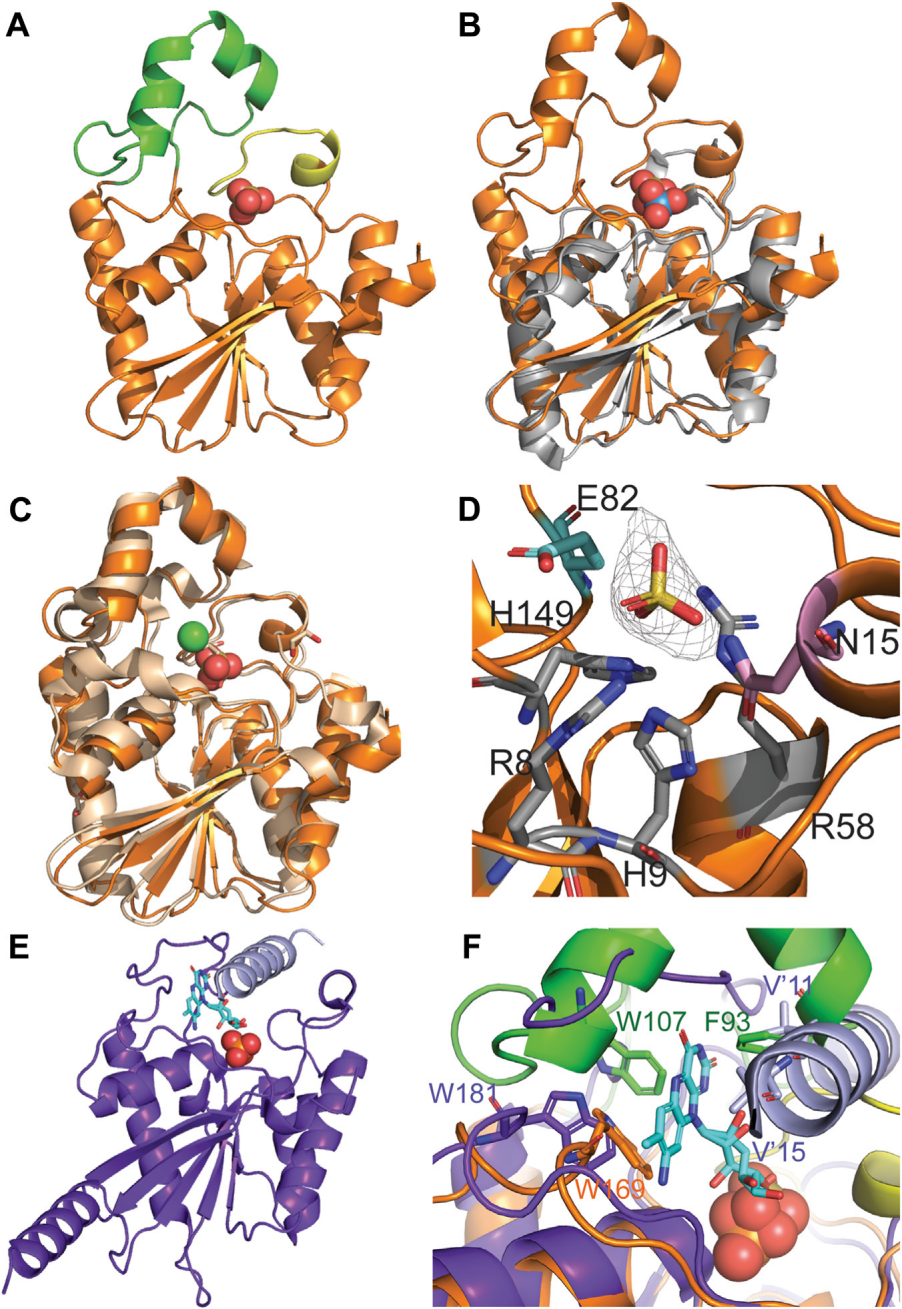
**Structure of HFP.***A*, HFP core domain is in *orange* and has two notable insertions shown in *green* (residues 85–120) and *yellow* (residues 14–24). Shown in the active site as the *gold* and *red spheres* is the bound sulfate. *B*, structure of HFP overlayed with *Escherichia coli* SixA. Overall structure of HFP is highly similar to other reported histidine family phosphatases. Shown in *gray* is the structure of the *E. coli* SixA (PDB: 1UJC) that represents the minimal core fold of the superfamily (25, 26). *C*, superposition of HFP (*orange*) with phosphoserine phosphatase of *Hydrogenobacter thermophilus* (PDB: 4IJ6), (*wheat*), the closest structural homologue as calculated by PDBeFold. The *green sphere* is a Cl ion bound to the 4IJ6 enzyme. *D*, the active site residues of HFP shown in *gray* (H9, R8, R58, and H149) are highly conserved across all histidine family phosphatases. H9 is the catalytic histidine that is phosphorylated and dephosphorylated over the course of catalysis. Residues in *pink* and *cyan*, N15 and E82, respectively, are also key residues for catalysis but are variable across known histidine family phosphatases. Together these highlighted residues comprise the “phosphate pocket” (26). Simulated annealing composite omit map with electron density of the sulfate ion contoured to 2 σ. *E*, structure of RosC, with monomer A in *purple* and the N-terminal helix of monomer B, which completes the active site, in *lavender*. Roseoflavin is shown as *cyan sticks*, and a phosphate ion is shown as *orange* and *red spheres*. *F*, overlay of the HFP active site with the RosC active site. Residues that may be important for ARAPDP binding by HFP (W169 in *orange* and W107 and F93 in *green*) are hypothesized based on the residues for roseoflavin binding (W181 in *purple* and V′11 and V′15 from the domain swapped N terminus of the dimer in *lavender*). ARAPDP, 5-amino-6-ribitylamino-pyrimidinedione 5′-phosphate; HFP, histidine family phosphatase.

The structure of HFP was determined to 2.04 Å by single anomalous dispersion using selenomethionine substituted protein. HFP, like all histidine family phosphatases, is composed of α/β/α sandwich where the central six stranded β-sheet has one antiparallel strand. Calculations in PDBeFold yielded 23 structures archived in the PDB with RMSD values less than 2.0 Å. Comparison of HFP with a representative of the minimal core fold of the superfamily, SixA (PDB: 1UJC) (25, 26), highlights the core α/β domain and illustrates two obvious insertions in the HFP structure (Fig. 7, *A* and *B*). These insertions are hypothesized to define substrate specificity (26). Using PDBeFold, the closest structural homolog is the phosphoserine phosphatase iPSP_1_ (PDB: 4IJ6), with an average RMSD of 1.6 Å for 211 α-carbons across all chains (Fig. 7*C*).

The reported biological assembly of members of the histidine family phosphatases range from monomers to dodecamers (25, 26, 27). HFP is a dimer in solution, as estimated by preparative gel filtration chromatography. The A and B chains crystallize in an orientation similar to the dimer observed in the iPSP_1_ homologue, whereas the C and D chains form these same contacts with chains in adjacent asymmetric units (Fig. S9). Calculations using PDBePISA estimated that interfaces between the A/B monomers and the C/D monomers were limited to between 900 and 950 Å^2^, which is at the lower end for supporting stable dimerization in solution. Similarly, iPSP_1_ was shown to be a dimer in solution and had a slightly higher interface area calculated of 1063 Å^2^.

The HFP active site, or “phosphate pocket,” contains four highly conserved residues characteristic of all histidine family phosphatases (26), R8, H9, R58, and H149, as determined by sequence and structure alignments (Fig. 7*D*). Based on published mechanisms for homologous phosphatases (26), it is logical to propose that residues R8, R58, and H149 form hydrogen bonds and electrostatic interactions with the bound phosphate group of the substrate, while H9 accepts the phosphate from the substrate, assisted by a proton donor, likely E82. Residues responsible for proton donation vary across families; however, E82 is situated to interact with bound substrate and overlays with the glutamate proton donor/acceptor of *Bacillus stearothermophilus* phosphatase PhoE (PDB; 1H2E) (28). Water hydrolysis of the phospho-H9 is assisted by the proton donor, E82, resetting the active site for further turnover. Finally, N15 of the phosphate pocket stabilizes substrate binding through hydrogen bonding as has been demonstrated across multiple histidine family phosphatases (26). There is significant density in the phosphate site that was modeled as sulfate derived from the crystallization conditions.

Recently, the structure of a HFP from roseoflavin biosynthesis was determined (29). Roseoflavin is an antibiotic generated from FMN by *Streptomyces* spp. and differs from riboflavin only by the replacement of the C8α methyl with a C8 exocyclic dimethyl-amine. The 8-demethyl-8-amino-riboflavin-5′-phosphate (AFP) phosphatase, called RosC, also dephosphorylates FMN with a 40-fold decrease in *k*_cat_/*K*_M_, but does not dephosphorylate ARAPDP as determined by an end point assay (29). The substrate specificity for AFP is derived from structural features that are not conserved in HFP: the insertions defined in Figure 7*A* (green and yellow), along with an N-terminal helical extension of RosC that provides an active site lid for the opposing monomer in a domain-swap (Fig. 7, *E* and *F*). The structure of RosC with AFP and a phosphate ion bound overlayed with the structure of HFP provide the ability to hypothesize which residues of HFP could be responsible for ARAPDP specificity. Phe93, Trp107, and Trp169 form a hydrophobic the cavity that would enclose the pyrimidinedione of ARAPDP, similar to hydrophobic pocket generated by Val11, Val15, and Trp181 in RosC.

### FMN phosphatase activity

EcYigB and RosC have been reported to exhibit FMN phosphatase activity. ^31^P-NMR was used to assess if HFP, the more specific of the two identified phosphatases for ARAPDP, was also able to dephosphorylate FMN (Fig. S10). Under the conditions used in this assay, FMN phosphatase activity by HFP was observed. The assay to determine ARAPDP phosphatase activity could not be modified to determine kinetic parameters for HFP, for comparison of relative specific activities for ARAPDP and FMN substrates. While kinetic parameters have been determined for nonspecific HAD phosphatases (9, 10), the experimental conditions do not translate for the temperatures required for turnover of hyperthermophile enzymes in the laboratory setting due to instability of pathway substrates (ex., NADPH) (30), and intermediates (epimerization of DARP and breakdown of ARAPDP) (7, 17).

## Discussion

All previously identified ARAPDP phosphatases are from the HAD superfamily of hydrolases (9, 10, 11). However, the HAD family of phosphatases are generally low fidelity and capable of indiscriminately dephosphorylating numerous substrates. For example, approximately 30% of HAD family phosphatases are capable of dephosphorylating FMN (31), including YigB, the ARAPDP phosphatase from *E. coli* (10). In an effort to identify a dedicated enzyme that completes the riboflavin biosynthetic pathway from the hyperthermophile *A. aeolicus,* which has a genome that is approximately one-third the size of *E. coli*, we screened the annotated phosphatases, including all HAD family hydrolases. We identified two enzymes capable of dephosphorylating ARAPDP, neither of which belong to the HAD superfamily of hydrolases.

IMP is reported as an IMP, which is a member of the phosphodiesterase family of enzymes and shares significant homology to the putative target for lithium therapy as a mood stabilizer (32). IMP is unable to facilitate riboflavin formation in combination with the rest of the pathway's enzymes from initial substrates GTP, NADPH, and Ru5P as it indiscriminately dephosphorylates NADPH and Ru5P.

HFP belongs to the histidine phosphatase superfamily, which is primarily composed of phosphatases, but a fraction of this family exhibit mutase activity. One such example is the glycolytic enzyme phosphoglycerate mutase (dPGM) (26, 33), which transfers the phosphate from the 2-OH of 2-phosphoglycerate to the 3-OH to generate 3-phosphoglycerate. The HFP sample under investigation in this study did not perform mutase activity and unexpectedly acted as a ribose 5-phosphaste isomerase, generating ribulose 5-phosphate. Ribose 5-phosphate isomerases (Rpi; EC 5.3.1.6) are hypothesized to use a mechanism analogous to that of phosphohexose isomerases in which a base residue facilitates the formation of a *cis*-enediol intermediate to interconvert the aldose and ketose sugar phosphates (34). There are two unrelated types of Rpis reported, RpiA (found in most organisms) and RpiB (found in some bacteria and parasitic protozoa) (34), neither of which have structural similarity to HFP (Fig. S11).

The two activities of the HFP sample are united by the context of the pathway, but are mechanistically distinct. Both the screened for phosphatase activity and the unexpected but advantageous isomerase activity facilitates riboflavin production. A strategy for increasing riboflavin production by microbial fermentation in *B. subtilis* has been to overexpress enzymes that generate the initial substrates of the pathway, including a D-ribose 5-phosphate (R5P)/Ru5P isomerase (RpiB) (35). Estimations of the relative intracellular concentrations of R5P and Ru5P indicate that R5P is in excess of Ru5P (35, 36, 37), so the isomerase converts the relatively abundant R5P into a direct riboflavin precursor. The apparent isomerase activity of this sample was highly alluring to attribute to HFP as it was present in samples purified by numerous orthogonal methods (heating, anion exchange chromatography, ammonium sulfate precipitation, affinity chromatography, and size-exclusion chromatography). However, four out of five randomly selected putative phosphatase samples demonstrated the isomerase activity and *E. coli* RpiA was detected in the sample by mass spectrometry, and so this second activity provides a cautionary tale for the over interpretation of contaminant activities. Regardless, in seeking a dedicated phosphatase capable of performing the dephosphorylation of ARAPDP in riboflavin biosynthesis, we succeeded in identifying two enzymes from novel phosphatase families that facilitate riboflavin formation. A recently characterized enzyme from roseoflavin biosynthesis gives credence that histidine family phosphatases can be evolved for flavin substrates. The HFP identified in this study has the appropriate specificity against substrates of riboflavin biosynthesis to be a dedicated phosphatase for this pathway.

## Experimental procedures

### Genomic screening and preparation of overexpression plasmids

Putative phosphatases (PPs) were identified by screening the published *A. aeolicus* genome (NCBI RefSeq assembly GCF_000008625.1, GenBank GCA_000008625.1) (38). Sequences annotated as phosphatases were filtered based on predicted molecular weight. Phosphatases with a molecular weight exceeding 40 kDa were excluded from further analysis. The amino acid sequences of the selected phosphatases were codon optimized for heterologous expression in *E. coli* and subcloned into pET20b for overexpression by Genscript. A stop codon was inserted prior to the C-terminal histidine tag so that proteins would be expressed in the native form. The *A. aeolicus* riboflavin biosynthetic pathway enzymes were cloned into pET29b plasmids also by Genscript for individual expression and purification of RibBA (3,4-dihydroxy-2-butananone-4-phosphate synthase/GTP cyclohydrolase II), RibD (deaminase/reductase), RibE (riboflavin synthase), and RibH (lumazine synthase). A previously reported enzyme that converts ARAPDP to ARAPD from *E. coli,* YigB (10), was cloned into pET28b by GenScript.

### Overexpression of enzymes

Plasmids containing the PPs and RibBA, riboflavin synthase, RibD, and lumazine synthase were individually transformed into *E. coli* BL21 (DE3) one shot competent cells (Thermo Fisher Scientific), and single colonies were used to make starter cultures for expression. For each PP, 100 ml LB broth with 200 μg/ml ampicillin was inoculated with 1 ml of overnight starter culture and grown at 37 °C with shaking (250 rpm) until the culture reached an *A*_600_ of 0.7. IPTG was added to a final concentration of 200 μM, and the flasks were moved to a 25 °C incubator for overnight shaking at 250 rpm. The following morning, cultures were centrifuged at 4347*g* for 15 min, and the pellets were frozen to −80 °C. For the production of RibBA, RibD, riboflavin synthase, and lumazine synthase, 10 ml of overnight starter culture was used to inoculate 1L of LB media containing 50 μg/ml kanamycin. The culture was grown at 37 °C with shaking until the *A*_600_ of 0.7 was reached. IPTG was added to a final concentration of 200 μM for each of the cultures producing RibBA, riboflavin synthase, and RibD, and the flasks were moved to 25 °C incubator for overnight incubation with shaking (250 rpm). To the lumazine synthase culture flask, IPTG was added to a final concentration of 1 mM, and the flask was incubated at 37 °C overnight with shaking (250 rpm). Overnight cultures were spun down the next morning at 4000*g* for 20 min at 4 °C. Pellets were stored in the −80 °C until ready for use.

### Lumazine synthase (RibH) purification

Lumazine synthase purification was based on a previously published protocol with minor changes (15). In short, the frozen cell mass from 1 L of culture was thawed in 50 mM Hepes pH 8.0, 150 mM NaCl and sonicated using the VWR sonicator fitted with a three-eighths inch horn. The sonication protocol consisted of five 30 s pulses and 30 s rest times at 50% amplitude. To prevent overheating of the sample, the sonication was performed in a metal beaker on ice. Cell debris was removed by centrifugation (20,000*g* for 20 min at 4 °C). The supernatant was heated to 85 °C for 10 min and subsequently chilled on ice for 5 min before centrifugation at 20,000*g* for 20 min at 4 °C. The resulting supernatant was loaded onto a HiLoad 16/600 Superose 6 size-exclusion column (Cytiva) preequilibrated with 50 mM Hepes pH 8.0, 150 mM NaCl, and 20% glycerol. The protein was eluted in the same buffer. Fractions containing lumazine synthase, as confirmed by SDS-PAGE, were pooled and concentrated using a nitrogen pressure cell with a Biomax 30 kDa Ultrafiltration membrane (Amicon). A concentration was determined by Bradford analysis, and the protein was aliquoted and stored in the −80 °C.

### Riboflavin synthase (RibE) purification

The frozen cell mass from 1 L of culture was thawed in 50 mM potassium phosphate pH 8.0, 20% glycerol and sonicated using the protocol described for lumazine synthase. Cell debris was removed by centrifugation (20,000*g* for 20 min at 4 °C). The supernatant was heated to 70 °C for 5 min, chilled 5 min on ice, and clarified by centrifugation at 20,000*g* for 20 min at 4 °C. The supernatant was diluted into 20 mM Tris pH 8.0, 20% glycerol by a factor of five before being loaded onto a 30Q anion exchange column (GE HealthCare) preequilibrated with the same buffer. The enzyme did not bind to the anion exchange resin, so the flow through was collected, concentrated using a nitrogen pressure cell with a Biomax 10 kDa Ultrafiltration membrane (Amicon), and applied to a 120 ml HiLoad Superdex 200 16/600 gel-filtration column (Cytiva) preequilibrated with 50 mM Hepes pH 8.0, 150 mM NaCl, 20% glycerol for isocratic elution. Fractions containing riboflavin synthase were pooled and concentrated using a nitrogen pressure cell with a Biomax 10 kDa Ultrafiltration membrane before being aliquoted and stored at −80 °C.

### RibBA purification

RibBA cell pellets were thawed and resuspended in 50 mM Hepes pH 8.0, 20% glycerol and sonicated using the protocol described for lumazine synthase. Cell debris was removed by centrifugation at 20,000*g* for 20 min at 4 °C. The supernatant was heated to 75 °C for 10 min, chilled 5 min on ice, and clarified by centrifugation at 20,000*g* for 20 min at 4 °C. The supernatant was diluted by a factor of five in 20 mM Tris pH 8.0, 20% glycerol and loaded onto a 30Q anion exchange column preequilibrated with the same buffer. The protein was eluted with a linear gradient of increasing a buffer of 20 mM Tris pH 8.0, 20% glycerol, and 1 M NaCl. Fractions containing RibBA were pooled, concentrated using a nitrogen pressure cell with a Biomax 30 kDa ultrafiltration membrane, and loaded on a 120 ml HiLoad 16/600 Superdex 200 size-exclusion column preequilibrated with 50 mM Hepes pH 8.0, 150 mM NaCl, 20% glycerol and the column was run isocratically. Fractions containing RibBA were pooled, concentrated using a nitrogen pressure cell with a Biomax 30 kDa ultrafiltration membrane, aliquoted, and stored at −80 °C.

### RibD purification

The frozen RibD cell mass from 1 L of culture was thawed in 50 mM Hepes pH 8.0, 20% glycerol, and sonicated using the protocol described for lumazine synthase. Cell debris was removed by centrifugation (20,000*g* for 20 min at 4 °C). The supernatant was heated to 70 °C for 5 min, chilled on ice, and clarified by centrifugation at 20,000*g* for 20 min at 4 °C. Less than 7.5 ml of supernatant was loaded directly onto a 120 ml HiLoad 16/600 Superdex 200 size-exclusion column preequilibrated with 50 mM Hepes pH 8.0, 150 mM NaCl, 20% glycerol, and the protein eluted with the same buffer. For protein preparations with higher volumes, the column was run multiple times and with smaller injection volumes of 1 to 1.5 ml to ensure resolution of eluted species. Fractions containing RibD were pooled and concentrated using a nitrogen pressure cell with a Biomax 30 kDa ultrafiltration membrane, and the concentration was determined by Bradford analysis. RibD was aliquoted and stored at −80 °C.

### YigB expression and purification

The overexpression plasmid containing the coding sequence for YigB was transformed in the ArcticExpress Competent Cells (Agilent) and expressed using manufacturer's guidelines. The cell pellet was stored at −80 °C until ready to lyse. The cell mass from 1 L of culture was thawed and resuspended in 20 mM Tris pH 8.0, 20% glycerol. The resuspended cells were sonicated using the protocol described for lumazine synthase. Cellular debris was removed by centrifugation at 20,000*g* for 30 min at 4 °C. The supernatant was loaded onto a Ni-affinity column (Chelating Sepharose Fast Flow from Cytiva) pre-equilibrated with 25 mM Tris pH 8.0, 300 mM NaCl and washed with same buffer. The YigB protein was eluted using a linear gradient of increasing imidazole to 500 mM. YigB was not stable in the high imidazole buffer, so the fractions containing YigB were confirmed by SDS-PAGE, pooled and immediately diluted with 50 mM Hepes pH 7.5 by a factor of two to decrease the imidazole concentration. The enzyme was concentrated using a nitrogen pressure cell with a Biomax 10 kDa Ultrafiltration membrane and loaded onto a 120 ml HiLoad Superdex 200 16/600 gel-filtration column (Cytiva) preequilibrated with 50 mM Hepes pH 7.5, 150 mM NaCl, and 20% glycerol. The YigB protein was eluted isocratically. Fractions containing YigB were pooled, concentrated using a nitrogen pressure cell with a Biomax 10 kDa ultrafiltration membrane, aliquoted, and stored at −80 °C until ready for use.

### Crude purification of PPs

The individual frozen cell masses from 50 ml of culture were thawed and resuspended in 5 ml 50 mM Hepes pH 8.0, 20% glycerol for sonication using the sonication protocol described for lumazine synthase. The lysate was centrifuged at 21,000*g* for 20 min. The supernatant was heated to 75 °C for 10 min before centrifugation at 21,000*g* for 20 min at 4 °C. The resulting supernatant was aliquoted and stored at −80 °C. The level of expression of the PPs and their relative purity was assessed by SDS-PAGE (Fig. S1).

### Large scale expression of putative phosphatase 11 (PP11), an IMP, and putative phosphatase 17 (PP17), a HFP

Overnight starter culture (10 ml) was used to inoculate 1 L of LB media containing 200 μg/ml ampicillin. The culture was grown at 37 °C with shaking (250 rpm) until the *A*_600_ of 0.7 was reached, and IPTG was added to a final concentration of 200 μM. Flasks containing PP11 culture were incubated at 25 °C overnight with shaking at 250 rpm, whereas flasks containing PP17 were incubated 30 °C overnight with shaking at 250 rpm. Overnight cultures were spun down the next morning at 4000*g* for 20 min at 4 °C. Pellets were stored in the −80 °C until ready for use.

### IMP purification for ^31^P-NMR and crystallization

IMP cell mass from 1 L of culture was thawed in 50 mM Hepes pH 8.0, 20% glycerol. The resuspension was sonicated using the protocol described for lumazine synthase and the lysate was centrifuged at 20,000*g* for 20 min at 4 °C. The supernatant was heated to 70 °C for 5 min, chilled on ice and clarified by centrifugation at 20,000*g* for 20 min at 4 °C. Supernatant was loaded onto a 120 ml HiLoad Superdex 200 16/600 gel-filtration column preequilibrated with 50 mM Hepes pH 8.0, 150 mM NaCl, 20% glycerol, and the column was run isocratically. Fractions containing IMP were pooled and concentrated using Amicon Ultra–15 Centrifugal Filters with a 10 kDa molecular weight cutoff. To get an accurate size estimate of IMP's molecular weight, the sample was applied to a 120 ml HiLoad 16/600 Superdex 75 prep grade size-exclusion column preequilibrated with 50 mM Hepes pH 8.0, 150 mM NaCl, 20% glycerol, and eluted with the same buffer. The molecular weight based on a standard curve generated from commercially available standard proteins is 65 kDa, making the enzyme a dimer in solution.

### HFP purification for ^31^P-NMR and crystallization

HFP cell mass from 1 L of culture was thawed in 50 mM Hepes pH 8.0, 20% glycerol. The resuspension was sonicated using the protocol described for lumazine synthase, and the lysate was centrifuged at 20,000*g* for 20 min at 4 °C. The supernatant was heated to 70 °C for 5 min and centrifuged at 20,000*g* for 20 min at 4 °C. The supernatant was diluted into 50 mM Hepes pH 8.0, 20% glycerol by a factor of four and loaded onto a 30Q anion exchange column preequilibrated in the same buffer. The protein was eluted using a linear gradient of 50 mM Hepes pH 8.0, 20% glycerol, 1 M NaCl over the course of 10 column volumes. Fractions containing HFP eluted between 100 and 300 mM NaCl. HFP containing fractions were pooled and concentrated using Amicon Ultra–15 Centrifugal Filters with a 10 kDa molecular weight cutoff. Protein was loaded onto a 120 ml HiLoad 16/600 Superdex 75 prep grade size-exclusion column preequilibrated with 50 mM Hepes pH 8.0, 150 mM NaCl, 20% glycerol, and the column was run isocratically. HFP containing fractions were pooled and concentrated using Amicon Ultra–15 Centrifugal Filters with a 10 kDa molecular weight cutoff to a concentration of 7 mg/ml per Bradford analysis. It is important to note, that HFP purified in this manner had considerable contamination, so HFP concentration was estimated based upon visual inspection of SDS-PAGE gels yielding an approximate concentration of 5 mg/ml HFP. HFP was aliquoted and frozen and stored in −80 °C until ready for use.

HFP samples used for NMR assays and ICPMS were purified more extensively using ammonium sulfate precipitation. In short, the cell mass from 1 L of liquid culture was resuspended in 50 mM Hepes pH 8, 150 mM NaCl. The sample was sonicated using the same sonication protocol described for lumazine synthase, and cell debris was removed by centrifugation at 20,000*g* for 20 min at 4 °C. The supernatant was heated to 55 °C for 5 min, cooled, and centrifuged at 20,000*g* for 20 min at 4 °C. Ammonium sulfate was added to a final concentration of 1 M (25% saturated solution), and the sample was centrifuged at 20,000*g* for 20 min at 4 °C. The supernatant was collected, and ammonium sulfate was added to a final concentration of 1.6 M (40% saturated solution). The sample was centrifuged at 20,000*g* for 20 min at 4 °C. The pellet was resuspended in 7.5 ml 50 mM Hepes pH 8, 20% glycerol, and loaded onto a 120 ml HiLoad 16/600 Superdex 75 prep grade size-exclusion column preequilibrated in 50 mM Hepes pH 8, 150 mM NaCl, and 20% glycerol, and fractions were eluted in the same buffer. Fractions containing HFP were confirmed by SDS-PAGE, pooled, and concentrated using an Amicon Ultra-15 Centrifugal Filter with a 10 kDa molecular weight cutoff.

### Purification of histidine-tagged HFP

In order to obtain an HFP sample of the highest possible purity to confirm the observed isomerase activity, a construct of HFP was generated in pET20b that had a C-terminal His tag. The tagged construct was expressed the same as the untagged construct. Frozen cell mass from 1 L of culture was thawed in 50 mM Hepes pH 7.5, 300 mM NaCl and lysed in the same manner as the untagged HFP. After sonication, lysate was centrifuged at 20,000*g* for 20 min at 4 °C. Supernatant was applied directly to a Ni-affinity column (Chelating Sepharose Fast Flow from Cytiva) preequilibrated with 50 mM Hepes pH 7.5, 300 mM NaCl, and 25 mM imidazole and washed with same buffer. Protein was eluted using a linear gradient of increasing imidazole up to 500 mM.

Fractions containing HFP were confirmed using SDS-PAGE, combined, and injected onto a 120 ml HiLoad 16/600 Superdex 75 prep grade size-exclusion column preequilibrated with 50 mM Hepes pH 8.0, 150 mM NaCl, 20% glycerol, and the column was run isocratically. Enzyme was concentrated and stored in the −80 °C until ready for use. Since the *A. aeolicus* enzyme is more heat stable than *E. coli* enzymes, the enzyme was further purified by heating 30 μl to 90 °C on a metal heat-block for 2 min (until the enzyme began to visibly precipitate). The sample was chilled on ice for 5 min before being centrifuged at 21,000*g* for 10 min. The supernatant was used for subsequent isomerization assays.

### Phosphatase activity screens

ARAPDP and DHBP were synthesized enzymatically by the addition of 6.7 μM RibBA, 23.9 μM RibD, 0.5 mM GTP, 1 mM Ru5P, and 0.5 mM NADPH in 500 μl of the reaction buffer (50 mM Hepes pH 8.0, 10 mM MgSO_4_). Because ARAPDP and ARAPD are sensitive to oxygen and will break down *via* a self-condensation reaction (17), a Schlenk line was used to generate an oxygen-free argon atmosphere. The sealed, anaerobic vessel was incubated at 37 °C overnight. The next morning, the resulting mixture containing ARAPDP and DHBP was divided among smaller flasks of 100 μl each. For the initial screen, a cocktail of PPs was added (a combination of four–five phosphatases). One flask contained the EcYigB positive control. All reaction vessels were returned to anaerobic conditions after the addition of the putative phosphatases. To account for differences in protein stability and species-dependent optimal temperature for enzymatic activity, flasks containing the *A. aeolicus* enzymes were incubated for 1 h in a 55 °C, while the EcYigB positive control was incubated for the same amount of time at 37 °C. After 1 hour, lumazine synthase and riboflavin synthase were added to each flask. All flasks were incubated at 55 °C for 2 h. The riboflavin content of the flasks was determined spectrophotometrically by 10-fold dilution of the reaction mixture in water and measuring the absorbance between 300 and 500 nm. Lumazine absorbs maximally at 410 nm, whereas riboflavin absorbs maximally at 445 nm. As determined previously (39, 40, 41), 470 nm was measured to determine riboflavin formation, allowing for discrimination of lumazine and riboflavin signals. Each flask was assayed individually. Once a given cocktail of phosphatases was confirmed to facilitate riboflavin formation, the phosphatases in that cocktail were assayed individually by the same protocol.

### One-pot assay

To 400 μl of reaction buffer (50 mM Hepes pH 8.0, 10 mM MgSO_4_), 7.6 μM RibBA, 10.8 μM RibD, 30.9 μM lumazine synthase, 33.3 μM riboflavin synthase, 1 mM GTP, 0.72 mM ribulose-5-phosphate (Ru5P) (Sigma-Aldrich), and 1 mM NADPH were added. The mixture was divided between two flasks of 250 μl each and 8.4 μM HFP and 8.8 μM IMP were added to each respective flask. Each vessel was sealed made anaerobic with an argon atmosphere using a Schlenk line and incubated at 55 °C for 4.5 h. The riboflavin content of the flasks was determined spectrophotometrically as in the screening protocol above. One-pot assays were completed in triplicate.

### Production, purification, and crystallization of selenomethionine substituted HFP

SeMet-HFP was produced by inhibiting methionine biosynthesis following previously published methodology (42). Notable alterations of the published procedure were that the LB broth (50 ml) was inoculated with 1 ml of overnight culture, and the culture was grown at 37 °C with shaking at 250 rpm to an *A*_600_ of 0.33. The cell culture was centrifuged at 5000*g* for 20 min at room temperature. The entire pellet was resuspended in 1 L of M9 minimal media with 200 μg/ml ampicillin. When the culture reached an *A*_600_ of 0.4 (37 °C, 250 rpm), the methionine biosynthesis pathway was inhibited by the addition of 100 mg each of L-lysine, L-phenylalanine, and L-threonine; 50 mg each of L-isoleucine, L-leucine, and L-valine; and 60 mg of D,L-selenomethionine per liter of culture and incubated for 15 min. IPTG was added to a final concentration of 200 μM, and the flask was moved to 30 °C with shaking at 250 rpm overnight. The next morning, the cells were harvested by centrifugation (4000*g*, 4 °C) and frozen at −80 °C. The 1.7 g cell mass was resuspended in 10 ml 50 mM Hepes pH 8.0, 150 mM NaCl, and 5 mM beta-mercaptoethanol (BME). The sample was sonicated using the same sonication protocol described for lumazine synthase, and cell debris was removed by centrifugation (20,000*g*, 20 min, 4 °C). The supernatant was heated to 55 °C for 5 min, cooled, and centrifuged (20,000*g*, 20 min, 4 °C). Ammonium sulfate was added to a final concentration of 1 M (25% saturated solution), and the sample was centrifuged (20,000*g*, 20 min, 4 °C). The supernatant was collected and ammonium sulfate added to a final concentration of 1.6 M (40% saturated solution). The sample was centrifuged (20,000*g*, 20 min, 4 °C). The pellet was resuspended in 8 ml 50 mM Hepes pH 8, 150 mM NaCl, 20% glycerol, 5 mM BME, and loaded onto a 120 ml HiLoad 16/600 Superdex 75 prep grade size-exclusion column preequilibrated in the same buffer. Fractions containing the protein of interest were confirmed by SDS-PAGE, pooled, and concentrated to 6 mg/ml using an Amicon Ultra-15 centrifugal filter with a 10 kDa molecular weight cutoff.

### IMP crystallization, X-ray diffraction data collection, and structure solution

Crystals were grown using the hanging drop method using drops composed of 1 μl enzyme solution (4.4 mg/ml IMP in 50 mM Hepes pH 8.0, 150 mM NaCl, and 20% glycerol) and 1 μl reservoir solution (75 mM Hepes pH 7.5, 70% 2-methyl-2,4-pentanediol [MPD]). The drop was allowed to come to vapor diffusion equilibrium over a 500 μl reservoir solution at room temperature. Prismatic crystals took more than 2 weeks to appear and were harvested after 19 days. The crystals were washed in reservoir solution and flash-cooled in liquid nitrogen. X-ray diffraction data were collected remotely at the Stanford Synchrotron Radiation Lightsource beamline 12-2 using a wavelength of 0.979 Å at 100 K. Oscillation images (1200; 0.15° each) were collected for a total of 180°. Each image had an exposure time of 0.1 s with 90% attenuation at a distance of 250 mm between the crystal and the Dectris Pilatus 6M pixel detector. The data were indexed and scaled with XDS (43) to 1.67 Å.

The structure was solved by molecular replacement using the Phaser-MR package in the Phenix software suite (44, 45). The molecular replacement search model used in Phaser was a monomer of 2PCR (PDB code) with waters removed. Phaser yielded a clear solution with a log likelihood gain of 7056 and translation function z-score of 65.2 with a dimer in the asymmetric unit. The 2PCR MR model had selenomethionine residues, which were replaced with methionine during refinement. Model building and refinement were performed with the software Coot (46) and phenix.refine (44). After the amino acid backbone was well refined, water and MPD molecules were added and manually verified in the subsequent refinements.

The A and B chains, which comprise one homodimer, have well-defined electron density, but the second homodimer, composed of chains C and D, has poorly defined density for secondary structure elements and missing density for a significant number of side chains (Fig. S12). There is missing density for two loops in the C and D chains, although this density is clearly present in the A and B chains. In sum, the packing of the homodimer of the C and D chains in the crystal lattice displays a higher degree of mosaicity. While the structure determined here and 2PCR are both in P21 with two homodimers in the asymmetric unit, they have different unit cell dimensions and the homodimers pack using different crystallization interfaces. All four chains of 2PCR are similarly well-resolved, but to roughly an Angstrom lower resolution overall compared to the structure determined here. The final model determined herein includes 992 residues in all four chains, two phosphates, eight magnesium ions, 323 waters, and nine MPD (a crystallant) molecules (Table S2). Statistics for data collection, structure solution, and refinement are found in Table 1. Ramachandran percentages were calculated using MolProbity (47). Calculations to compare this structure to previously published phosphatases were performed using PDBeFold (48, 49) and calculations for determining stable interfaces were performed in PDBePISA (50).

**Table 1:** X-ray crystallographic statistics

	IMP	HFP-SeMet	HFP
Data collection			
PDB Accession Code	9MMI		9MMH
Wavelength (Å)	0.979	0.979	0.979
Space group	P2_1_	P3_1_2	P3_1_2
Unit cell dimensions	a = 65.19, b = 72.91, c = 119.25, β = 90.03°	a = 110.08, b = 110.08, c = 257.43, γ = 120°	a = 110.01, b = 110.01, c = 256.65, γ = 120°
Resolution range (Å)	37.68–1.75 (1.78–1.75)	39.12–2.10 (2.13–2.1)	39.02–2.04 (2.07–2.04)
R_merge_	0.081 (0.888)	0.098 (0.854)	0.149 (2.717)
R_pim_	0.034 (0.410)	0.039 (0.415)	0.034 (0.637)
CC1/2	0.998 (0.775)	0.998 (0.659)	0.999 (0.927)
Total reflections	789,589 (33,783)	1,026,120 (26,913)	2,387,220 (111,982)
Unique reflections	110,871 (5264)	105,231 (4567)	115,401 (5580)
Redundancy	7.1 (6.4)	9.8 (5.9)	20.7 (20.1)
Completeness (%)	98.5 (95.0)	98.9 (87.6)	99.9 (98.8)
Mean I/sigma(I)	11.3 (2.0)	13.6 (2.1)	17.2 (2.1)
Selenomethionine Phasing			
Selenium Sites (#)		36	
FOM		0.392	
Overall Score		61 ± 7	
Refinement			
Resolution range (Å)	37.68–1.75 (1.78–1.75)		39.02–2.04 (2.07–2.04)
R_cryst_	0.1823 (0.2588)		0.1900 (0.2828)
R_free_	0.2087 (0.2894)		0.2252 (0.3148)
Reflections used in refinement	110,785 (7455)		115,252 (7877)
Reflections used for R-free	2007 (134)		2006 (140)
Number of nonhydrogen atoms	8188		7125
Macromolecules	7776		6680
Ligands	90		55
Solvent	322		390
Protein residues	992		812
RMS (bonds)	0.012		0.013
RMS (angles)	1.252		1.281
Average B-factor	39.65		38.02
Ramachandran favored (%)	97.23		96.89
Ramachandran allowed (%)	2.67		3.11
Ramachandran outliers (%)	0.10		0

### HFP crystallization, X-ray diffraction data collection, and structure solution

SeMet and native HFP crystals were prepared identically using sitting drop vapor-diffusion equilibrium. Crystallization drops were prepared by combining 0.5 μl of enzyme solution (50 mM Hepes pH 8, 150 mM NaCl, 20% glycerol, and 5 mM BME) was combined with 0.5 μl of reservoir solution (100 mM Tris–HCl pH 8.5, 2 M ammonium sulfate) and allowed to come to vapor-diffusion equilibrium with 40 μl of reservoir solution. Crystals were visible after 4 days of incubation at 25 °C, and were harvested after 12 days. Crystals were washed in reservoir solution with increasing glycerol concentrations of 0%, 10%, and 20%, respectively, and flash cooled in liquid nitrogen. X-ray diffraction data were collected remotely at the Stanford Synchrotron Radiation Lightsource beamline 12-2 at a wavelength of 0.9793 Å. For the SeMet data, 1200 oscillation images with a delta of 0.15° were collected for a total of 180°. Each image had an exposure time of 0.1 s with 90% attenuation at a distance of 350 mm between the crystal and the Dectris Pilatus 6M pixel detector. The data were indexed and scaled with XDS (43) to 2.10 Å. For the native HFP dataset, oscillation images (1800; 0.2° each) were collected for a total of 360° using a wavelength of 0.9793 Å at 100 K. Each image had an exposure time of 0.1 s with 80% attenuation at a distance of 300 mm between the crystal and the Dectris Pilatus 6M pixel detector. The data were indexed and scaled with XDS (43) to 2.05 Å.

The HFP structure was initially determined by selenium single anomalous dispersion using Phenix AutoSol. Substructure determination identified the location of 36 selenium atoms, and the program Autobuild (Phenix) generated an initial protein model. Alignment of selenium atoms with the sidechains of methionine residues was performed, such that six selenium atoms were determined per six methionines in each of the four monomers in the asymmetric unit. A rudimentary refinement using phenix.refine (44) with manual adjustments to the Se-Met model were made in Coot resulting in an initial model with *R*_work_ of 0.1970 and *R*_free_ of 0.2117. (46). The native structure was solved by molecular replacement using the initial Se-Met protein model and the Phaser-MR package in the Phenix software suite (44, 45). Phaser yielded a clear solution with a log likelihood gain of 35,630 and translation function z-score of 144.2 with a tetramer in the asymmetric unit. Model building and refinement were performed with the software Coot (46) and phenix.refine (44). The final model includes all 203 residues in all four chains, 11 sulfate anions from the crystallization conditions, and 395 modeled water molecules. Statistics for data collection, structure solution, and refinement are found in Table 1. A comparison of structures and calculation of RMSD values were performed using PDBeFold (48). Structure figures were generated in PyMOL (PyMOL Molecular Graphics System, version 2.0, Schrodinger, LLC). Calculations for determining stable interfaces were performed in PDBePISA (50) and Ramachandran outliers were calculated using MolProbity (47).

### ^31^P-NMR experiments

All ^31^P-NMR experiments were carried out in triplicate using a Bruker Avance III HD (500 MHz) spectrometer equipped with a Prodigy CryoProbe at 298 K. Briefly, 512 scans were recorded using a zgpg30 pulse sequence. Samples were generated in the assay reaction buffer (50 mM Hepes pH 8.0, 10 mM MgSO_4_). Chemical shifts were determined by combining assay reaction buffer (2 ml) with 15 μl 90 mM Ru5P (0.67 mM final concentration) or solid GTP and NADPH (0.5 mM final concentration) in separate vials. The three different solutions were divided into 500 μl aliquots. To each aliquot, 1.4 μM YigB, HFP, or IMP, or the same volume of enzyme buffer was added (negative control). HFP, IMP, and the buffer negative control were incubated at 55 °C, whereas YigB was incubated at 37 °C. After 1 h, the enzyme was removed using an Amicon Ultra-0.5 ml Centrifugal Filter with a 10 kDa molecular weight cutoff. The flowthrough (400 μl) was combined with 100 μl D_2_O. Spectra of pure standards of GTP, NADPH, and Ru5P at the same concentrations and using the same buffer conditions provided reference chemical shifts. The product of the HFP reaction (ribose 5-phosphate) was confirmed by ^31^P-NMR (pulse sequence pgzg30) and ^13^C-NMR (pulse sequence uDEFT).

To determine the reversibility of the isomerase reaction catalyzed by HFP, 1.4 μM HFP was incubated with either 0.67 M ribose 5-phosphate or 0.67 M ribulose 5-phosphate for 1 h at 55 °C. HFP was removed using an Amicon Ultra-0.5 ml Centrifugal Filter with a 10 kDa molecular weight cutoff, and D_2_O added to make a 10% solution. The ^31^P-NMR was recorded after 20 min. The solutions were moved to a 55 °C incubator for 2 h then assayed again. Controls for ribulose 5-phosphate and ribose 5-phosphate stability were prepared the same way but in the absence of HFP.

The ability HFP to dephosphorylate FMN was assessed by ^31^P-NMR by combining 0.6 mM FMN in reaction buffer (50 mM Hepes pH 8.0, 10 mM MgSO_4_) with 1.4 μM YigB or HFP and incubated at 37 °C and 55 °C, respectively, for 1 h before running NMR. A negative control without enzyme was also incubated at 55 °C for 1 h to ensure that any detected dephosphorylation was due to enzymatic activity and not breakdown at high temperatures.

### ^13^C-NMR experiments

All ^13^C-NMR experiments were carried out once on Bruker Avance III HD (500 MHz) spectrometer equipped with a Prodigy CryoProbe at 298 K with 1024 scans and udeft pulse sequence. ^13^C-labeled ribulose 5-phosphate was prepared as previously described (6): 50 μl of a 126 mM ^13^C-ribulose 5-phosphate stock was diluted in 850 μl 50 mM Hepes pH 8.0, 10 mM MgSO_4_, and 100 μl D_2_O for a final concentration of 6.3 mM ribulose 5-phosphate. This sample was divided into two aliquots. The first aliquot was combined with 50 μl HFP (final concentration of 7.6 μM) to produce an NMR spectra containing an equilibrium of Ru5P and R5P ^13^C-NMR signals, and the second was combined with 50 μl of 50 mM Hepes pH 8.0, 150 mM NaCl, 20% glycerol (the HFP storage buffer) to generate an enzyme free negative control that provides the spectra of ^13^C- ribulose 5-phosphate. Unlabeled ribose 5-phosphate (81 mM in 90% H_2_O 10% D_2_O) was subjected to ^13^C-NMR to provide the chemical shifts of the ribose 5-phosphate carbons using ^13^C natural abundance.

### Inductively coupled plasma mass spectrometry experiments

IMP is annotated as being an IMP, which is metal dependent. Therefore, ICPMS was employed to unambiguously determine the identities of the metals observed in the electron density of the active site. Storage buffer (50 mM Hepes pH 8.0, 150 mM NaCl, and 20% glycerol), 88 μM IMP, and 84 μM HFP were diluted 10-fold in 4% nitric acid. Samples were loaded onto a Shimadzu ICPMS-2030 equipped with a mini torch and an AS-10 autosampler. Helium was the collision gas to break apart possible polyatomic interferences.

## Data availability

Crystallographic coordinates and structure factors are deposited in the Protein Data Bank. Accession codes are found in Table 1. All other data are presented here or in the supplement, which will be provided upon request (audrey.lamb@utsa.edu).

## Supporting information

This article contains supporting information.

## Conflict of interest

The authors declare that they have no conflicts of interest with the contents of this article.
