# Supplementary material for: Identification of the phosphatase essential for riboflavin biosynthesis in Aquifex aeolicus
Source: J Biol Chem. 2025 Mar 25;301(5):108443. doi: 10.1016/j.jbc.2025.108443 (PMC12051542; doi:10.1016/j.jbc.2025.108443)
Supplement: Supporting information [file mmc1.docx]

**Identification of the phosphatase that catalyzes the essential substrate dephosphorylation step of riboflavin biosynthesis in *Aquifex aeolicus***

Zoe A. Hoffpauir and Audrey L. Lamb

Department of Chemistry, University of Texas at San Antonio, San Antonio, TX

**Supporting Information**

**Contents**

Script S1: Python script for identifying consensus sequences Page S-2

Table S1: Enzymes used in this study Page S-4

Table S2: Model components for IMP structure Page S-5

Table S3: Model components for HFP structure Page S-5

Figure S1: SDS-PAGE gel showing expression of phosphatases Page S-6

Figure S2: SDS-PAGE gel of purified IMP and HFP Page S-7

Figure S3: ^31^P-NMR of prolonged incubation samples demonstrates Page S-8

GTP dephosphorylation

Figure S4: ^13^C-NMR to confirm Ru5P/R5P isomerization Page S-9

Figure S5: Equilibrium mixture of ribose and ribulose 5-phosphate Page S-10

established by HFP sample

Figure S6: His-tagged HFP sample isomerization of Ru5P Page S-11

Figure S7: Isomerase activity by ^31^P-NMR Page S-12

Figure S8: Mass Spectrometry Confirmation of RpiA Page S-13

Figure S9: HFP asymmetric composition and biological assembly Page S-14

Figure S10: FMN phosphatase activity by ^31^P-NMR Page S-15

Figure S11: Structural comparison of HFP to RpiA and RpiB Page S-16

Figure S12: Electron density comparison in IMP monomers Page S-17

**Script S1. Python Script for identifying consensus sequences.** The published proteins for *Aquifex aeolicus ^14^* (text file) were imported into Microsoft Excel and saved as a CSV file with each protein being on its own row.

#import packages

import pandas as pd
#define functions
def check_IV(sequence):
    a=1
    hits=[]
    for i in range(len(sequence)-5):
        if sequence[i]=='G' and sequence[i+1]=='D' and sequence[i+5]=='D':
            hits.append(a)
        a=a+1
    for i in range(len(sequence)-6):
        if sequence[i]=='G' and sequence[i+1]=='D' and sequence[i+6]=='D':
            hits.append(a)
        a=a+1
    for i in range(len(sequence)-1):
        if sequence[i]=='D' and sequence[i+1]=='D':
            hits.append(a)
        a=a+1
    return sorted(hits)

def mark_pos(Hits):
    if len(Hits)>0: return 1
    else: return 0

def check_I(sequence):
    hits=[]
    a=1
    for i in range(len(sequence)-6):
        if sequence[i]=='D' and sequence[i+2]=='D' and ((sequence[i+4]==('T' or 'V')) and (sequence[i+5])==('L'or'I')):
            hits.append(a)
        a=a+1
    return hits

def mark_Ipos(Hits):
    if len(Hits)>0: return 1
    else: return 0

#construct dataframe
df= pd.read_csv(path to data, names=['ProteinID'])

df[['ProteinID','Sequence']]=df['ProteinID'].str.split(']',1,expand=True)

df['Sequence']=[str(ele) for ele in df.Sequence]
df['IV_Hits']= [check_IV(ele1) for ele1 in df.Sequence]
df['IV_Positive']= [mark_pos(ele1) for ele1 in df.IV_Hits]
df=df.loc[df.IV_Positive==1]

df['I_Hits']= [check_I(ele1) for ele1 in df.Sequence]
df['NumberI']=[len(ele) for ele in df.I_Hits]
df['I_Positive']= [mark_pos(ele1) for ele1 in df.I_Hits]
df['Sum']=df.IV_Positive+df.I_Positive

df=df.loc[df.Sum==2]

#create CSV with hits
df.to_csv('Aacuratedhits.csv')

**Table S1: Enzymes used in this study**

| **Name** | **Genomic Annotation** | **Code** |
| --- | --- | --- |
| RibBA | bifunctional 3,4-dihydroxy-2-butanone-4-phosphate synthase/GTP cyclohydrolase II | WP_010880177.1 |
| RibE | riboflavin synthase | WP_010881107.1 |
| RibD | bifunctional diaminohydroxyphosphoribosyl-aminopyrimidine deaminase/ 5-amino-6-(5-phosphoribosylamino) uracil reductase | WP_010880032.1 |
| RibH | 6,7-dimethyl-8-ribityllumazine synthase (lumazine synthase) | WP_010880027.1 |
| PP1 | dUTP diphosphatase | WP_010880090.1 |
| PP2 | phosphatidylglycerophosphatase A | WP_010880153.1 |
| PP3 | serine/threonine-protein phosphatase | WP_010880576.1 |
| PP4 | inorganic diphosphatase | WP_010881004.1 |
| PP5 | phosphohistidine phosphatase SixA | WP_010880736.1 |
| PP6 | bifunctional oligoribonuclease/PAP phosphatase NrnA | WP_010881055.1 |
| PP7 | lipid A 1-phosphatase LpxE | WP_010881106.1 |
| PP8 | nucleoside triphosphate pyrophosphatase | WP_010881116.1 |
| PP10 | bifunctional phosphoribosyl-AMP cyclohydrolase/ phosphoribosyl-ATP diphosphatase HisIE | WP_010881285.1 |
| PP11 (IMP) | inositol monophosphatase family protein | WP_010881296.1 |
| PP12 | histidine phosphatase family protein | WP_010881302.1 |
| PP13* | undecaprenyl-diphosphatase UppP | WP_010881444.1 |
| PP14 | acylphosphatase | WP_164930633.1 |
| PP15 | 1 Ppx/GppA phosphatase family protein | WP_164930648.1 |
| PP16 | UDP-2,3-diacylglucosamine diphosphatase LpxI | WP_164930695.1 |
| PP17 (HFP) | histidine phosphatase family protein | WP_164930753.1 |
| PP18 | RdgB/HAM1 family non-canonical purine NTP pyrophosphatase | WP_010880078.1 |
| PP20 | low molecular weight phosphatase family protein | WP_010880379.1 |
| PP21 | acireductone synthase | WP_010881291.1 |
| PP22 | HAD family hydrolase | WP_010880861.1 |
| PP23 | HAD hydrolase-like protein | WP_010880597.1 |
| PP24 | HAD hydrolase-like protein | WP_164930629.1 |
| PP25 | nucleoside hydrolase | WP_010880610.1 |
| * = The protein did not express adequately for subsequent screening, see Figure S1 gel.  ** = PP9 and PP19 were removed because they were open reading frame duplicates from the RefSeq and GenBank predicted proteomes. | | |

| **Table S2: IMP Structure Components** |  |
| --- | --- |
| Total Residues | 992 |
| Total Waters | 323 |
| Chain A | 260 Residues: 3-262  1 Phosphate  2 Magnesium Atoms  4 MPD |
| Chain B | 261 Residues: 3-263  1 Phosphate  2 Magnesium Atoms  4 MPD |
| Chain C | 233 Residues: 4-29, 42-74, 79-160, 167-258  2 Magnesium Atoms  1 MPD |
| Chain D | 238 Residues: 4-34, 42-160, 168-255  2 Magnesium Atom |

| **Table S3: HFP Structure Components** |  |
| --- | --- |
| Total Residues | 812 |
| Total Waters | 395 |
| Chain A | 203 Residues: 1-203 |
| Chain B | 203 Residues: 1-203 |
| Chain C | 203 Residues: 1-203 |
| Chain D | 203 Residues: 1-203 |
| Chain E | 11 sulfates |

**Figure S1: SDS-PAGE showing expression of phosphatases.** Boxes highlight presence of putative phosphatase. Samples noted with * were diluted 1:10 prior to loading on to the gel for clarity as undiluted sample overloaded the lane. PP9 and PP19 were removed because they were open reading frame duplicates from the RefSeq and GenBank predicted proteomes.

**Figure S2: SDS-PAGE gel showing purified IMP and HFP used for assays.** Note that there are two faint bands visible in the HFP sample between 48 and 63 kDa. Neither of these bands are the *E. coli* Rpi contaminants responsible for the observed isomerase activity of the sample. The molecular weights of *E. coli* RpiA and RpiB are ~22,860 and 16,073 Da, respectively. Because HFP is close in molecular weight to RpiA, the contaminant band may not be visible by SDS-PAGE.

**Figure S3**: **^31^P-NMR of** **prolonged incubation samples demonstrates GTP dephosphorylation.** Overnight incubation of enzymes with GTP does show that GTP is dephosphorylated by HFP and IMP after prolonged incubation at 55˚ C. In particular, note the formation of the peak at 2.1 ppm which is free phosphate (highlighted by the gray box). A free phosphate peak is absent in the GTP standard, which was subjected to identical temperature and duration of incubation.

**Figure S4**: **^13^C-NMR to confirm Ru5P/R5P isomerization.** Top black trace shows labeled Ru5P and R5P generated by ^13^C-Ru5P isomerization by HFP. Blue trace shows chemical shifts of carbons in ^13^C-Ru5P. Unlabeled R5P was used to determine chemical shifts of the carbons in R5P (bottom red trace).

**Figure S5: Equilibrium mixture of ribose and ribulose 5-phosphate established by HFP sample**. Integration of ^31^P peaks showing an equilibrium mixture of 39.7% ketose (ribulose) and 60.3% aldose (ribose) stereoisomer 5-phosphates established by HFP sample during a 10 min incubation at room temperature. Calculation of equilibrium concentrations of Ru5P and R5P was performed in MestReNova.

**Figure S6:** **His-tagged HFP sample isomerization of Ru5P**. After affinity chromatography, gel filtration, and heating to 90 °C for 2 minutes (until protein began to visibly fall out of solution), HFP sample still isomerizes Ru5P to R5P, but it takes longer for the 40:60 equilibrium to be reached. The top panel shows the sample after being incubated for 1 hour with Ru5P vs 18 hours. Only after a prolonged incubation time is equilibrium reached, suggesting significantly less contaminant Rpi present.


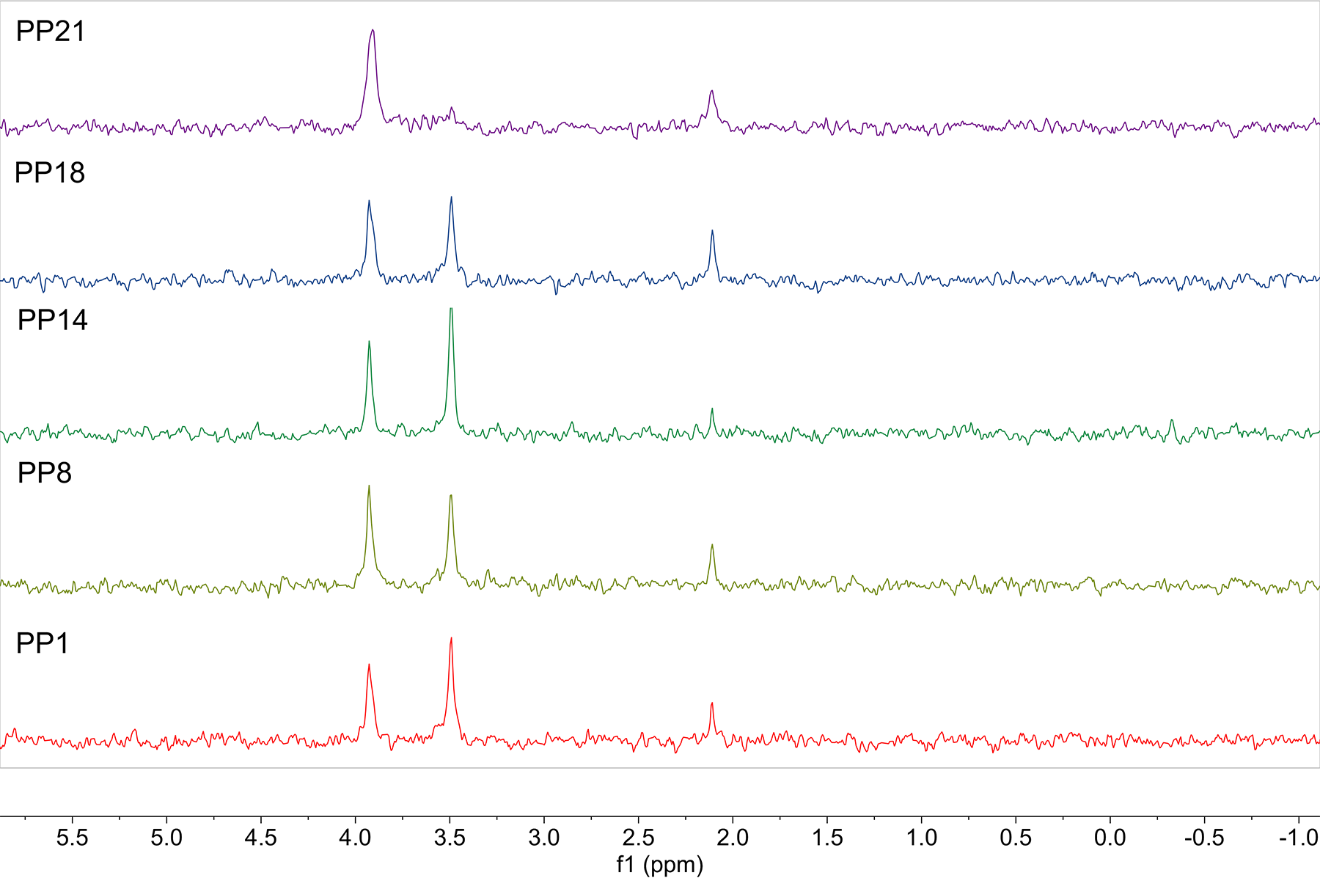


**Figure S7: Isomerase activity by ^31^P-NMR.** Four out of five randomly selected putative phosphate samples demonstrated isomerase activity, as evident from the new peak at 3.5 ppm.


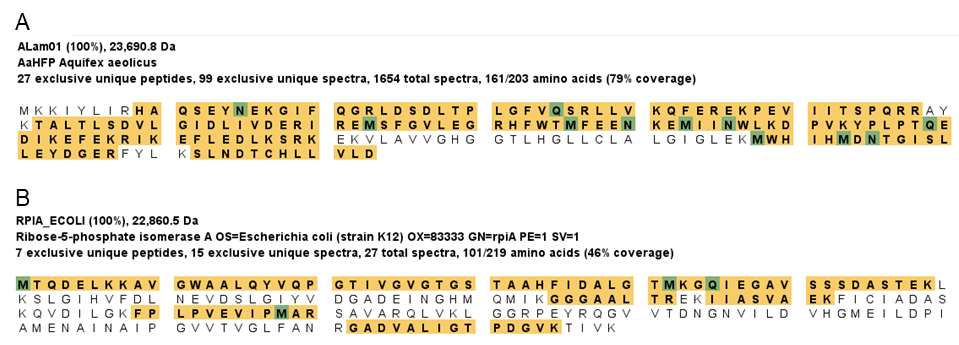


**Figure S8. Mass spectrometry confirmation of RpiA** Sequence coverage from mass spectrometry analysis. **A**, HFP (*Aquifex aeolicus*); **B**, ribose-5-phosphate isomerase A (*Escherichia coli*). Sequences highlighted in gold were detected after applying the criteria described below. Detected modifications of amino acids are indicated in green: M, methionine oxidation; Q, glutamine deamidation; N asparagine deamidation. [Note: the modifications were detected on a percentage of residues—i.e., they are not all modified.]

***MS Method***. Digests were analyzed by HPLC-electrospray ionization tandem mass spectrometry on a Thermo Scientific Orbitrap Fusion Lumos mass spectrometer using a data-dependent acquisition scan strategy. Mascot (v2.8.3; Matrix Science, London UK) was used to search the spectra against a combination of the following databases: UniProt_E_coli_ref; a “local” database that includes the sequences of recombinant and target proteins; common contaminants (not including Bos taurus proteins). Cysteine carbamidomethylation was set as a fixed modification and methionine oxidation and deamidation of glutamine and asparagine were considered as variable modifications; trypsin was specified as the proteolytic enzyme, with two missed cleavages allowed. The Mascot search results were imported into Scaffold (version 5.3.3, Proteome Software Inc., Portland, OR): peptide threshold, 95%; protein threshold, 99%; minimum peptides, 2. The settings used resulted in a protein-level FDR of ~0%.

**Figure S9: HFP asymmetric unit and biological assembly. A**. HFP crystals contained 4 chains in the asymmetric unit colored as follows: A, dark orange, B, bright orange, C, wheat, D, gray. Chains A and B form the dimer while chains C and D form dimers with adjacent unit cells. **B**. The biological assembly has high homology to PDB:4IJ6, shown in cyan, and the active sites are highlighted by the green spheres.

**Figure S10: FMN phosphatase activity by ^31^P-NMR.** ^31^P-NMR indicates that both EcYigB, in agreement with previously published reports, and HFP dephosphorylate FMN.

S-15

**Figure S11: Structural comparison of HFP (orange) to RpiB (green, PDB: 3K7S) and RpiA (pink, PDB: 1LK7).**

**Figure S12:** **Electron density comparison in IMP monomers**. The density for residues 151-160, 183-186, and 203-298 is shown for both the A and D chains. The 2Fo-Fc map is contoured to 1.5 σ.
